# On Modern Progress of Ophthalmic Medicine and Surgery

**Published:** 1894-03-24

**Authors:** Robert Brudenell Carter

**Affiliations:** Consulting Ophthalmic Surgeon to St. George's Hospital


### On Modern Progress in Ophthalmic Medicine and Surgery.

By Robert Brudenell Carter, F.R.C.S., Consulting Ophthalmic Surgeon to St. George's Hospital.

The "flap-operation" for the extraction of cataract, when completely successful, left nothing to be desired as regards the excellence of its results; but its advantages were diminished by a large percentage of complete or of comparative failures. It might have been selected as an appropriate illustration of that well-known maxim of political economy which declares that "all learners spoil a portion of raw material." The skill necessary for its dexterous performance could only be obtained by considerable practice, and it gave occasion to Beer's often quoted saying that a cataract operator would destroy a hatful of eyes before he cured one. In this country, even in the most skilful hands, about 15 per cent. of the eyes operated upon perished utterly from suppuration, and in about 25 per cent. more the visual results were but indifferent, leaving only 60 per cent. of good or passable cures. The chances in favour of the patient were little more than even, and cases which terminated unfavourably were often attended by severe and long-continued suffering. Hence, it was usually considered inexpedient to operate as long as the sight admitted of being altered for the worse; and extraction was commonly deferred until both eyes were practically blind.

The failures of flap extraction depended partly upon the actual difficulty of the operation itself, upon the difficulty, that is, of accomplishing what was desired and nothing more; partly upon the position and nature of the wound, and partly upon risks of septic inflammation, which, in those days, were neither understood nor guarded against. As already described, the operator steadied the movable eyeball against his knife by two fingers of his disengaged hand. If the pressure exerted by these fingers were excessive, the opaque lens was liable to be ejected from the eye, together with a quantity of vitreous humour, at the moment of completing the incision; while, if the pressure were insufficient, the eyeball was likely to be rotated inwards before the knife, so that the inner edge of the cornea would pass out of view towards the inner canthus, the point of counter-puncture would be concealed, and its precise position, as well as that of the completed incision, would be matters of great uncertainty. When the incision was correctly placed, entirely within, but only just within, the true corneal tissue, and having the horizontal meridian of the cornea as its base-line, not only was the size of the resulting opening sufficient to allow of the easy exit of the lens, but the central portion of the cornea, through which vision had to be obtained, was not liable to distortion of its curvature by the contraction incidental to healing. If these conditions were not fulfilled; if, for example, the counter-puncture were made too far forward from the corneal margin; or if, by faulty position of the blade, the line of incision as a whole were placed too low; in either case the exit of the lens was likely to be difficult, and to be attended by loss of vitreous; and also in either case the central curvature of the cornea was liable to be implicated in, and injuriously modified by, the effects of cicatricial contraction. If vitreous were lost, it continued to escape after closure of the lids, and it lifted the iris into the incision, so that the pupil was dragged upwards, sometimes to such an extent as to require a secondary operation; while distortion of the corneal curvature, when produced, was a permanent and irremediable cause of very defective vision.

Assuming the flap operation to have been correctly and successfully performed, the issue was still dependent upon prompt healing of the external wound and upon the absence of inflammation. In order to promote the attainment of these conditions, it was customary to close the eyelids with strips of court plaster, and not to open them for five or six days. During this time the progress of the case could be judged of, to some extent, by the state of the lids and by the character of the discharge, if any, which exuded from between them. In favourable cases the lids remained free from swelling, and the discharge consisted only of a little mucus. In unfavourable cases the swelling of the lids became considerable, and the discharge assumed a purulent character. In neither condition was any treatment adopted; it being felt that, if all were well, inspection of the wound might involve risk, and that, if inflammation had commenced, it might be aggravated, and could not be relieved, by interference. In cases of the latter kind it was usual, when the lids at last were opened, to find complete sloughing of the cornea, and for this to be followed by suppuration of the whole contents of the eyeball. In this country, as already stated, suppuration occurred, even in the most skilful hands, in about 15 per cent. of the cases operated upon; but in Germany, where, from the poor feeding of the peasantry, cataract was far more common than in England, and where, from the same cause, there was far greater liability to necrotic change, the losses from suppuration were much more considerable, while in Russia, where adverse conditions prevailed in still greater intensity, they were more considerable still. In the Moscow eye hospital, on large numbers, suppuration occurred in 45 per cent. of the cases.

Although English surgeons in those days resigned themselves to a total loss of 15 per cent. of their operations as to an unavoidable evil, the still greater losses which occurred in Germany directed the thoughts of Albrecht von Graefe to the possibility of diminishing them. His first step, in this direction, was to endeavour to discover the starting point of the mischief; and, for this purpose, he departed from the old usage of leaving the lids closed, and began to open them, and to examine the eye, every day. He soon found it possible to divide the failures into three classes: namely, those in which the corneal flap underwent sloughing because the portion still attached was unable to maintain it; those in which the corneal flap sloughed because its edge fell away from contact with that from which it had been severed; and those in which the inflammation commenced as iritis. This iritis first appeared opposite the flap; and, although we now know that it must usually have been of a septic character, it was attributed by von Graefe to bruising of the iris fibres by the passage of the lens as it was pressed through the circular and uninterrupted pupil.

